# Intermittent Catheterization: The Devil Is in the Details

**DOI:** 10.1089/neu.2017.5413

**Published:** 2018-04-01

**Authors:** Kathleen Christison, Matthias Walter, Jean-Jacques J.M. Wyndaele, Michael Kennelly, Thomas M. Kessler, Vanessa K. Noonan, Nader Fallah, Andrei V. Krassioukov

**Affiliations:** ^1^ International Collaboration on Repair Discoveries (ICORD), Faculty of Medicine, University of British Columbia, Vancouver, British Columbia, Canada.; ^2^ Department of Urology, University of Antwerp, Antwerp, Belgium.; ^3^ Department of Urology, Carolinas Medical Center, Charlotte, North Carolina.; ^4^ Neuro-Urology, Spinal Cord Injury Center & Research, University of Zürich, Balgrist University Hospital, Zürich, Switzerland.; ^5^ Rick Hansen Institute, Vancouver, British Columbia, Canada.; ^6^ Division of Neurology, Department of Medicine, Faculty of Medicine, University of British Columbia, Vancouver, British Columbia, Canada.; ^7^ Division of Physical Medicine and Rehabilitation, Department of Medicine, Faculty of Medicine, University of British Columbia, Vancouver, British Columbia, Canada.; ^8^ G.F. Strong Rehabilitation Center, Vancouver, British Columbia, Canada.

**Keywords:** Cochrane review, intermittent catheterization, neurogenic lower urinary tract dysfunction, spinal cord injury, urinary tract infection

## Abstract

During the last few years, the international community debated urinary tract infection and re-use of catheters when managing neurogenic lower urinary tract dysfunction (NLUTD) among individuals with spinal cord injury (SCI). In this respect, the 2014 Cochrane review by Prieto and colleagues, “Intermittent catheterisation for long-term bladder management,” became one of the leading documents that captured the minds and attention of clinicians around the world. Although numerous countries had switched to single-use catheters for management of NLUTD following SCI, the opinion that was expressed in the 2014 Cochrane review had a strong influence on healthcare providers and agencies to recommend re-use of catheters. However, many clinicians have expressed concern regarding the conclusions in the 2014 Cochrane review by Prieto and colleagues. We therefore conducted an independent appraisal of the data and analyses presented in the review. Our appraisal identified crucial discrepancies of data extraction and analyses within the review. In appraisal to that of Prieto and colleagues' review, our analysis revealed a trend to favor single over multiple use of catheters. After addressing our concerns to Cochrane's acting Editor-in-Chief, the most recent version of the 2014 Cochrane review was withdrawn from publication.

## Introduction

During the last few years, the international community has engaged in strong debates on urinary tract infection (UTI) and re-use of catheters during the management of neurogenic lower urinary tract dysfunction (NLUTD) among individuals with spinal cord injury (SCI). The most frequent complication of intermittent catheterization is UTI.^1^ There is no universally accepted definition of UTI in individuals with NLUTD, particularly in those with SCI. While UTI is an evolving term that varies between organizations, its potentially devastating effect is of no argument. UTI is costly to both the healthcare system and to individuals and can result in frequent hospitalization, sepsis, and even death.^2^

In this respect, the Cochrane 2014 systematic review “Intermittent catheterisation for long-term bladder management” by Prieto and colleagues, became one of the leading documents that captured the minds and attention of clinicians around the world.^3,4^

Although the authors did identify numerous limitations and risk for bias within the trials included in their review (p.8), they came to the conclusion that “there is still no convincing evidence that the incidence of UTI is affected by use of aseptic or clean technique, coated or uncoated catheters, single (sterile) or multiple-use (clean) catheters, self-catheterisation or catheterisation by others, or by any other strategy” (p.2). This has influenced clinicians' opinions and recommendations on intermittent catheterization over the last few years.

However, upon closer inspection of the review, we are confident that this conclusion requires revision.

## Methods

We completed a thorough appraisal of the 2014 Cochrane review by Prieto and colleagues, focusing on 1) correct data selection and extraction of all 31 trials included, that is, 13 randomized controlled trials (RCTs) and 18 cross-over RCTs (Table 1), 2) use of an up-to-date definition of UTI, and 3) statistical appropriateness and correctness of all 39 analyses.

**Table 1.** Trials Included in the 2014 Cochrane Review (*n* = 31)

*No.*	*Year*	*Author*	*Trial design*	*Journal, year, (month), volume, issue, pages*
1	2013	Chartier-Kastler et al.	Cross-over RCT	J. Urol. 2013 Sep;190(3):942–7.
2	2013	Costa et al.	Cross-over RCT	Spinal Cord 2013 Oct;51(10):772–5.
3	2013	Leek et al.	Cross-over RCT^**c**^	Neurourol. Urodyn. 2012;32(6):759–60.
4	2013	Moore et al.	Cross-over RCT^**c**^	Neurourol. Urodyn. 2013;32(6):760–1.
5	2012	Denys et al.	Cross-over RCT^d^	Spinal Cord 2012 Nov;50(11):853–8.
6	2011	Cardenas et al.	RCT	PM R 2011 May;3(5):408–17.
7	2011	Chartier-Kastler et al.	Cross-over RCT	Spinal Cord 2011 Jul;49(7):844–50.
8	2011	Domurath et al.	Cross-over RCT	Spinal Cord 2011 Jul;49(7):817–21.
9	2010	Sarica et al.	Cross-over RCT^a,d^	Eur. J. Phys. Rehabil. Med. 2010 Dec;46(4):473–9.
10	2009	Cardenas and Hoffman	RCT	Arch. Phys. Med. Rehabil. 2009 Oct;90(10):1668–71.
11	2009	Witjes et al.	RCT	J. Urol. 2009 Dec;182(6):2794–8.
12	2007	Biering-Sorensen et al.	Cross-over RCT	Scand. J. Urol. Nephrol. 2007;41(4):341–5.
13	2006	Leriche et al.	Cross-over RCT	Prog. Urol. 2006 Jun;16(3):347–51.
14	2006	Moore et al.	RCT	Clin. Rehabil. 2006 Jun;20(6):461–8.
15	2005	De Ridder et al.	RCT	Eur. Urol. 2005 Dec;48(6):991–5.
16	2005	Taweesangsuksalul et al.	Cross-over RCT^d^	J. Thai Rehabil. Med. 2005;15(2):113–8.
17	2003	Day et al.	RCT	Urol. Nurs. 2003 Apr;23(2):143–7, 158.
18	2003	Vapnek et al.	RCT	J. Urol. 2003 Mar;169(3):994–8.
19	2002	Fera et al.	RCT^d^	Braz. J. Urol. 2002;28(1):50–6.
20	2001	Fader et al.	Cross-over RCT^b,d^	BJU Int. 2001 Sep;88(4):373–7.
21	2001	Giannantoni et al.	Cross-over RCT^d^	J. Urol. 2001 Jul;166(1):130–3.
22	2001	Mauroy et al.	Cross-over RCT^a,d^	Ann. Urol. (Paris) 2001 Jul;35(4):223–8.
23	2001	Pascoe and Clovis	Cross-over RCT^d^	Br. J. Nurs. 2001 Mar 8–21;10(5):325–9.
24	2001	Schlager et al.	Cross-over RCT	Pediatrics 2001 Oct;108(4): E71.
25	1999	Pachler and Frimodt-Moller	Cross-over RCT	BJU Int. 1999 May;83(7):767–9.
26	1997	Prieto-Fingerhut et al.	RCT	Rehabil. Nurs. 1997 Nov–Dec;22(6):299–302.
27	1996	Sutherland et al.	RCT	J. Urol. 1996 Dec;156(6):2041–3.
28	1995	Duffy et al.	RCT	J. Am. Geriatr. Soc. 1995 Aug;43(8):865–70.
29	1993	Moore et al.	Cross-over RCT	Rehabil. Nurs. 1993 Sep–Oct;18(5):306–9.
30	1993	Quigley and Riggin	RCT	Rehabil. Nurs. 1993 Jan–Feb;18(1):26–9, 33.
31	1992	King et al.	RCT	Arch. Phys. Med. Rehabil. 1992 Sep;73(9):798–802.

Overall, 31 trials, comprising 13 parallel group randomized controlled trials (RCTs) and 18 cross-over RCTs, were included for qualitative analysis in the 2014 Cochrane review. The crossover RCTs either had two (*n* = 15, unmarked), three (*n* = 2, marked^a^), or four arms *(n* = 1, marked^b^).

^c^ Reports were only available as congress or meeting abstracts.

^d^ Eight of 31 trials were classified as not providing data in a format that could be used in meta-analysis by Prieto and colleagues. However, two trials, i.e., #9 (Sarica et al. 2010) and #21 (Giannantoni et al. 2001) provided data that could have been included in meta-analysis.

## Results

We identified four main concerns with the 2014 Cochrane review by Prieto and colleagues:

### First concern (data selection)

Of the 31 trials included in the 2014 Cochrane review, 2 were published only as conference abstracts (Table 1). Further, Prieto and colleagues reported “Eight of the 31 trials did not provide data in a format that could be used in meta-analysis” (p.8). However, 2 of these 8 trials (Table 1) did provide data that could have been included in meta-analysis.

### Second concern (data extraction)

**FIG. 1.**
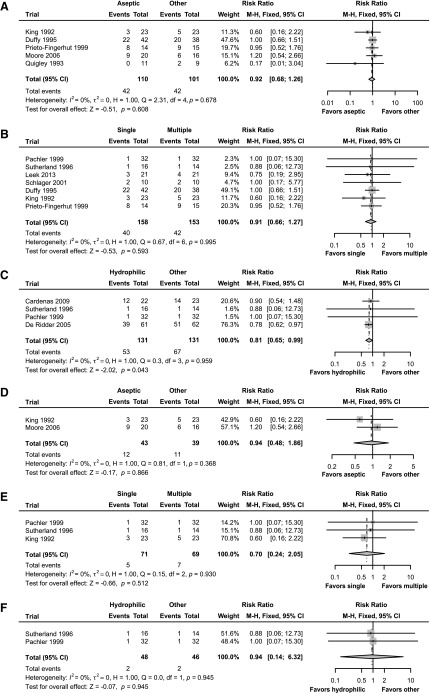
A new perspective on analyses 1.2, 2.2, and 3.2 of the 2014 Cochrane review. **(A)** Analysis 1.2: Aseptic versus other technique. All five trials (UTI defined according to Cochrane) from the original Cochrane meta-analysis were included. Applying the Mantel-Haenszel (M-H) method (fixed effect model) for meta-analysis did not show any significant difference (*p* = 0.608) between aseptic versus other technique with regards to the incidence of UTI. (**B)** Analysis 2.2: Single versus multiple use of catheters. Seven of eight trials (UTI defined according to Cochrane) from the original Cochrane meta-analysis were included. Moore et al. 2013 was excluded because of missing data. Applying the M-H method (fixed effect model) for meta-analysis did not show any significant difference (*p* = 0.593) between single versus multiple use of catheters with regards to the incidence of UTI. **(C)** Analysis 3.2: Hydrophilic versus other catheters. Four of five trials (UTI defined according to Cochrane) from the original Cochrane meta-analysis were included. Moore et al. 2013 was excluded because of missing data. Applying the M-H method (fixed effect model) for meta-analysis did show a significant difference (*p* = 0.043) between hydrophilic versus other catheters with regards to the incidence of UTI. De Ridder et al. 2005 is the only trial providing significant evidence favoring hydrophilic over another type of catheter. This trial is also the only one with a high number of participants (*n* = 123) and long investigation period (12 months). The authors of the Cochrane review refrained from deriving a summary estimate because of the heterogeneity among the trials and attrition bias. However, we did not find an issue with heterogeneity (see results above). (**D)** Analysis 1.2: Aseptic versus other technique. After adjustment was made regarding the UTI definition (according to the Infectious Diseases Society of America [IDSA]), only two trials were included in this analysis. Applying the M-H method (fixed effect model) for meta-analysis did not show any significant difference (*p* = 0.866) between aseptic versus other technique with regards to the incidence of UTI. Given the small number of participants (*n* = 82) and unclear duration of investigation (between 4 weeks and a minimum of 7 weeks), no final conclusion can be drawn. **(E)** Analysis 2.2: Single versus multiple use of catheters. After adjustment was made regarding the UTI definition (according to the IDSA), only three trials were included in this analysis. Applying the M-H method (fixed effect model) for meta-analysis did not show any significant difference (*p* = 0.512) between single versus multiple use of catheters with regards to the incidence of UTI. Given the small number of participants (*n* = 140) and short duration of investigation (maximum 8 weeks), no final conclusion can be drawn. **(F)** Analysis 3.2: Hydrophilic versus other catheters. After adjustment was made regarding the UTI definition (according to the IDSA), only two trials were included in this analysis. Applying the M-H method (fixed effect model) for meta-analysis did not show any significant difference (*p* = 0.945) between hydrophilic versus other catheters with regards to the incidence of UTI. Given the small number of participants (*n* = 94) and short duration of the investigation (maximum 8 weeks), no final conclusion can be drawn. CI, confidence interval.

Upon close inspection five types of disagreements were observed:

1. Data were mislabeled;
2. Extracted data did not match data from original trial;
3. Data were extracted in a method not consistent with convention;
4. Data were in a form that could not be used in meta-analyses; and
5. Data were not originally extracted, although eligible (Supplementary Table 1 and Fig. 1; see online supplementary material at http://www.liebertpub.com).

For example, in “*Analysis 2.2”* data from six of the eight trials provided by Prieto and colleages were not consistent with originally published data. At times, it appears that the authors extracted data from an original trial correctly, but placed the data under an incorrect heading or the data were presented only partially, that is, one instead of both arms of the cross-over trial was reported.

### Third concern (symptomatic UTI definition)

Although the review was published in 2014, the UTI definition was taken from an outdated 1992 National Institute on Disability and Rehabilitation Research (NIDRR) consensus statement.^5^ Prieto and colleagues also chose to accept definitions for symptomatic UTI as reported in the trials reviewed (p.3). As a result, heterogeneous definitions of symptomatic UTI were included in analysis. However, at the time the Cochrane review was conducted, the Infectious Diseases Society of America (IDSA) 2009 Consensus Statement had already provided the most up-to-date and comprehensive definition of UTI, which specifically covered catheter-associated UTI (Table 2).^6^

**Table 2.** Comparison of Definitions of Symptomatic Urinary Tract Infection

*(NIDRR) – 1992^5^*	(*IDSA) – 2009^6^*
■ Bacteriuria (≥100 bacteria/mL of urine) with tissue invasion and resultant tissue response with signs and/or symptoms.	■ CA-UTI in patients with indwelling urethral, indwelling suprapubic, or intermittent catheterization is defined by the presence of symptoms or signs compatible with UTI with no other identified source of infection along with ≥1000 cfu/mL of ≥1 bacterial species in a single catheter urine specimen or in a midstream voided urine specimen from a patient whose urethral, suprapubic, or condom catheter has been removed within the previous 48 h.
■ Signs and symptoms: Leukocytes in the urine generated by the mucosal lining; discomfort or pain over the kidney or bladder, or during urination; onset of urinary incontinence; fever; increased spasticity; autonomic hyperreflexia; cloudy urine with increased odor; malaise, lethargy, or sense of unease.	■ Data are insufficient to recommend a specific quantitative count for defining CA-UTI in symptomatic men when specimens are collected by condom catheter.
	■ CA-ASB should not be screened for except in research studies evaluating interventions designed to reduce CA-ASB or CA-UTI and in selected clinical situations, such as in pregnant women.
	■ CA-ASB in patients with indwelling urethral, indwelling suprapubic, or intermittent catheterization is defined by the presence of ≥100,000 cfu/mL of ≥1 bacterial species in a single catheter urine specimen in a patient without symptoms compatible with UTI.
	■ CA-ASB in a man with a condom catheter is defined by the presence of ≥100,000 cfu/mL of ≥1 bacterial species in a single urine specimen from a freshly applied condom catheter in a patient without symptoms compatible with UTI.
	■ Signs and symptoms compatible with CA-UTI include new onset or worsening fever, rigors, altered mental status, malaise, or lethargy with no other identified cause; flank pain; costovertebral angle tenderness; acute hematuria; pelvic discomfort; and in those whose catheters have been removed, dysuria, urgent or frequent urination, or suprapubic pain or tenderness.
	■ In patients with spinal cord injury, increased spasticity, autonomic dysreflexia, or sense of unease are also compatible with CA-UTI.
	■ In the catheterized patient, pyuria is not diagnostic of CA-bacteriuria or CA-UTI. The presence or absence of odorous or cloudy urine alone should not be used to differentiate CA-ASB from CA-UTI or as an indication for urine culture or antimicrobial therapy.
	■ The presence, absence, or degree of pyuria should not be used to differentiate CA-ASB from CA-UTI.
	■ Pyuria accompanying CA-ASB should not be interpreted as an indication for antimicrobial treatment.
	■ The absence of pyuria in a symptomatic patient suggests a diagnosis other than CA-UTI.

CA-ASB, catheter-associated asymptomatic bacteriuria; CA-UTI, catheter-associated urinary tract infection; cfu/mL, colony-forming units per milliliter; IDSA, Infectious Diseases Society of America; NIDRR, National Institute on Disability and Rehabilitation Research; UTI, urinary tract infection.

### Fourth concern (data analysis)

Of all 39 analyses from the Cochrane review (Supplementary Table 1), 20 consisted of only one trial. However, according to the *Cochrane Handbook for Systematic Reviews of Interventions* Version 5.1.0 at least two trials are required for meta-analysis.^7^ Of the remaining 19 analyses that compared two or more trials, only four analyses included data that matched the originally published data. There were also inconsistencies with subtotals and totals displayed within the forest plots of numerous analyses. In “*Analysis 3.2*,” Prieto and colleagues “chose not to derive a summary estimate because of heterogeneity amongst the trials and the problem of attrition bias.” Surprisingly, although the authors noted the same issue with heterogeneity in *“Analysis 2.2”* and stated, “We decided not to derive a summary…,” they still included subtotal and total summaries in the forest plot of this analysis (pp.73–74).

Following careful re-evaluation of all trials that were included in the Cochrane review, which led to necessary corrections (data selection, extraction, and use of up-to-date definition for UTI), we computed all 19 analyses comprising at least two trials according to the *Cochrane Handbook*.^7^

In contrast to the review by Prieto and colleagues, we found that *“Analysis 2.2”* exhibits a trend (albeit small) toward the single use (sterile) of catheters, and *“Analysis 3.2”* significantly favors hydrophilic catheters (Fig. 1). Further, when we applied the up-to-date (IDSA) definition of symptomatic UTI, at least 50% of trials from the 2014 Cochrane review *“Analysis 2.2 and 3.2”* had to be excluded due to outdated UTI definitions (Fig. 1).

## Discussion

Given the presented evidence, we strongly believe that the statement made in the Cochrane review: “…there is still no convincing evidence that the incidence of UTI is affected…” by any of the established intermittent catheterization techniques has to be corrected. When analyses were performed (after data correction) using the 2014 Cochrane review definitions for UTI, no difference was found between single versus multiple use of catheters. However, the use of hydrophilic versus other catheters demonstrated a significantly lower incidence of UTI. Further, when applying the up-to-date IDSA definition of UTI, a trend favoring single versus multiple use of catheters was detected, which is in contrast to the conclusion of Prieto and colleagues. Until evidence can confidently demonstrate that multiple use is as safe as single use of catheters, healthcare providers should advocate a single use of catheters in individuals with SCI, especially considering that catheter cleaning is a major issue because there is no standardized and universally accepted cleaning method that would be the prerequisite for multiple use of catheters. A future and more homogeneous systematic review is necessary to identify evidence that has accumulated since 2014. If analyses remain inconclusive, further high-quality RCTs with adequate number of participants and trial duration, are necessary to derive conclusive results.

## Supplementary Material

Supplemental data

Supplemental data
