# Supplementary material for: Intermittent Catheterization: The Devil Is in the Details
Source: J Neurotrauma. 2018 Apr 1;35(7):985–9. doi: 10.1089/neu.2017.5413 (PMC5865623; doi:10.1089/neu.2017.5413)
Supplement: Supplemental data [file Supp_Table1.pdf]

Supplementary Data

SUPPLEMENTARY TABLE 1. OVERVIEW OF DISCREPANCIES BETWEEN DATA FROM ORIGINAL TRIALS AND 2014 COCHRANE REVIEW ANALYSES

	Data from 2014 Cochrane Review		*Data from original trials*		*Discrepancies in data extraction*
Analysis 1.1					
Trials	Aseptic technique (number with asymptomatic bacteriuria/all subjects)	Clean/other aseptic technique (number with asymptomatic bacteriuria/all subjects)	Aseptic technique (number with asymptomatic bacteriuria/all subjects)	Clean/other aseptic technique (number with asymptomatic bacteriuria/all subjects)	
Moore 2006	7/16	9/20	**9/20**	**7/16**	**1. Data mislabeled.**
Day 2003	0/6	2/5	0/6	2/5	None.
Analysis 1.2					
Trials	Aseptic technique (number with symptomatic UTI/all subjects)	Clean/other aseptic technique (number with symptomatic UTI/all subjects)	Aseptic technique (number with symptomatic UTI/all subjects)	Clean/other aseptic technique (number with symptomatic UTI/all subjects)	
Moore 2006	6/16	9/20	**9/20**	**6/16**	**1. Data mislabeled.**
Prieto-Fingerhut 1999	9/14	8/15	**8/14**	**9/15**	**2. Data did not match original trial.**
Duffy 1995	20/38	22/42	**22/42**	**20/38**	**1. Data mislabeled.**
Quigley 1993	1/11	1/9	**0/11**	**2/9**	**2. Data did not match original trial.**
King 1992	5/23	3/23	**3/23**	**5/23**	**1. Data mislabeled.**
Analysis 1.3					
Trials	Aseptic technique (weeks to onset of symptomatic UTI; mean [SD])	Clean/other technique (weeks to onset of symptomatic UTI; mean [SD])	Aseptic technique (weeks to onset of symptomatic UTI; mean [SD])	Clean/other technique (weeks to onset of symptomatic UTI; mean [SD])	
Moore 2006	16; −4.6(3.3)	20; −4.3(1.5)	**20; 3.6(1.3)**	**16; 3.0(2.4)**	**2. Data did not match original trial.**
Duffy 1995	42; −3.11(3.12)	38; −3.5(3.02)	**42; 3.49(3.03)**	**38; 3.11(3.13)**	**1. Data mislabeled.**
King 1992	23; −1.1(0.87)	23; −1.25(1.05)	**23; 1.26(1.06)**	**23; 1.1(0.87)**	**1. Data mislabeled.**
Analysis 2.1					
Trials	Single use (number with asymptomatic bacteriuria/all subjects)	Multiple use (number with asymptomatic bacteriuria/all subjects)	Single use (number with asymptomatic bacteriuria/all subjects)	Multiple use (number with asymptomatic bacteriuria/all subjects)	
Leek 2013	4/12	4/10	**9/21**	**7/21**	**3. Data extracted in a method not consistent with convention.**
Schlager 2001	10/10	9/10	10/10	9/10	None.
Pachler 1999	13/32	16/32	13/32	16/32	None.
Sutherland 1996	NA	NA	**2/16**	**3/14**	**5. Data not originally extracted.**
Moore 1993	17/33	16/33	**N/A**	**N/A**	**4. Data in a form that could not be used in meta-analysis.**
King 1992	NA	NA	**10/23**	**10/23**	**5. Data not originally extracted.**

(*continued*)

Supplementary Table 1. (Continued)

	Data from 2014 Cochrane Review		*Data from original trials*		*Discrepancies in data extraction*
Analysis 2.2					
Trials	Single use (number with symptomatic UTI/all subjects)	Multiple use (number with symptomatic UTI/all subjects)	Single use (number with symptomatic UTI/all subjects)	Multiple use (number with symptomatic UTI/all subjects)	
Leek 2013	2/12	2/10	**3/21**	**4/21**	**3. Data extracted in a method not consistent with convention.**
Moore 2013	2/45	3/45	**N/A**	**N/A**	**4. Data in form that could not be used in meta-analysis.**
Schlager 2001	2/10	2/10	2/10	2/10	None.
Pachler 1999	1/32	1/32	1/32	1/32	None.
Prieto-Fingerhut 1999	9/14	8/15	**8/14**	**9/15**	**2. Data did not match original trial.**
Sutherland 1996	3/16	4/14	**1/16**	**1/14**	**3. Data extracted in a method not consistent with convention.**
Duffy 1995	20/38	22/42	**22/42**	**20/38**	**1. Data mislabeled.**
King 1992	5/23	3/23	**3/23**	**5/23**	**1. Data mislabeled.**
Analysis 2.3					
Trials	Single use (weeks to onset of symptomatic UTI; mean [SD])	Multiple use (weeks to onset of symptomatic UTI; mean [SD])	Single use (weeks to onset of symptomatic UTI; mean [SD])	Multiple use (weeks to onset of symptomatic UTI; mean [SD])	
Duffy 1995	42; –3.11(3.12)	38; –3.5(3.02)	**42; 3.49(3.03)**	**38; 3.11(3.13)**	**1. Data mislabeled.**
King 1992	23; –1.1(0.87)	23; –1.25(1.05)	**23; 1.26(1.06)**	**23; 1.1(0.87)**	**1. Data mislabeled.**
Analysis 3.1					
Trials	Catheter A (number with asymptomatic bacteriuria/all subjects)	Catheter B (number with asymptomatic bacteriuria/all subjects)	Catheter A (number with asymptomatic bacteriuria/all subjects)	Catheter B (number with asymptomatic bacteriuria/all subjects)	
Pachler 1999	13/32	16/32	13/32	16/32	None.
Sutherland 1996	NA	NA	**2/16**	**3/14**	**5. Data not originally extracted.**
Analysis 3.2					
Trials	Catheter A (number with symptomatic UTI/all subjects)	Catheter B (number with symptomatic UTI/all subjects)	Catheter A (number with symptomatic UTI/all subjects)	Catheter B (number with symptomatic UTI/all subjects)	
Moore 2013	2/45	3/45	**N/A**	**N/A**	**4. Data in form that could not be used in meta-analysis.**
Cardenas 2009	12/22	14/23	12/22	14/23	None.
De Ridder 2005	39/61	51/62	39/61	51/62	None.
Pachler 1999	1/32	1/32	1/32	1/32	None.
Sutherland 1996	3/16	4/14	**1/16**	**1/14**	**3. Data extracted in a method not consistent with convention.**

(*continued*)

Supplementary Table 1. (Continued)

	Data from 2014 Cochrane Review		*Data from original trials*		*Discrepancies in data extraction*
Analysis 3.4					
Trials	Catheter A (number with urethral trauma, bleeding or macroscopic hematuria/all subjects)	Catheter B (number with urethral trauma, bleeding or macroscopic hematuria/all subjects)	Catheter A (number with urethral trauma, bleeding or macroscopic hematuria/all subjects)	Catheter B (number with urethral trauma, bleeding or macroscopic hematuria/all subjects)	
Cardenas 2011	14/105	6/114	14/105	6/114	None.
Leriche 2006	0/29	5/29	0/29	5/29	None.
De Ridder 2005	38/55	32/59	38/55	32/59	None.
Giannantoni 2001	N/A	N/A	**0/18**	**2/18**	**5. Data not originally extracted.**
Pachler 1999	1/32	1/32	1/32	1/32	None.
Analysis 3.8					
Trials	Catheter A (number reporting overall satisfaction/all subjects)	Catheter B (number reporting overall satisfaction/all subjects)	Catheter A (number reporting overall satisfaction/all subjects)	Catheter B (number reporting overall satisfaction/all subjects)	
Moore 2013	35/48	42/48	35/48	42/48	None.
Witjes 2009	70/81	87/88	**72/81**	**87/88**	**2. Data did not match original trial.**
De Ridder 2005	9/25	7/33	9/25	7/33	None.
Analysis 3.9					
Trials	Catheter A (mean overall satisfaction; mean [SD])	Catheter B (mean overall satisfaction; mean [SD])	Catheter A (mean overall satisfaction; mean [SD])	Catheter B (mean overall satisfaction; mean [SD])	
Cardenas 2011	105; 9.3(1.4)	114; 8.6(1.3)	105; 9.3(1.4)	114; 8.6(1.3)	None.
Leriche 2006	29; 7(2.3)	29; 5.7(2.5)	29; 7(2.3)	29; 5.7(2.5)	None.
Sutherland 1996	17; 4.1(2.5)	16; 3.6(2.1)	**17; 3.3(3.0)**	**16; 3.9(2.1)**	**3. Data extracted in a method not consistent with convention.**
Analysis 3.10					
Trials	Catheter A (number reporting preference/all subjects)	Catheter B (number reporting preference/all subjects)	Catheter A (number reporting preference/all subjects)	Catheter B (number reporting preference/all subjects)	
Leriche 2006	19/29	10/29	19/29	10/29	None.
De Ridder 2005	9/25	7/33	**N/A**	**N/A**	**4. Data in form that could not be used in meta-analysis.**
Pachler 1999	15/32	11/32	15/32	11/32	None.
Analysis 3.16					
Trials	Catheter A (mean ease of insertion; mean [SD])	Catheter B (mean ease of insertion; mean [SD])	Catheter A (mean ease of insertion; mean [SD])	Catheter B (mean ease of insertion; mean [SD])	
Cardenas 2011	105; 9.2(1.6)	114; 8.6(1.6)	105; 9.2(1.6)	114; 8.6(1.6)	None.
Sutherland 1996	17; 3.6(2.6)	16; 3.6(2.5)	**17; 2.7(2.4)**	**16; 4.2(2.6)**	**3. Data extracted in a method not consistent with convention.**

(*continued*)

Supplementary Table 1. (Continued)

	Data from 2014 Cochrane Review		*Data from original trials*		*Discrepancies in data extraction*
Analysis 4.1					
Trials	Shorter length (number reporting ease of handling/all subjects)	Standard length (number reporting ease of handling/all subjects)	Shorter length (number reporting ease of handling/all subjects)	Standard length (number reporting ease of handling/all subjects)	
Costa 2013	62/81	73/81	62/81	73/81	None.
Chartier-Kastler 2011	27/30	26/30	**28/30**	**26/30**	**2. Data did not match original trial.**
Domurath 2011	34/36	28/36	34/36	28/36	None.
Analysis 4.2					
Trials	Shorter length (number reporting ease of insertion/all subjects)	Standard length (number reporting ease of insertion/all subjects)	Shorter length (number reporting ease of insertion/all subjects)	Standard length (number reporting ease of insertion/all subjects)	
Costa 2013	58/81	71/81	58/81	71/81	None.
Chartier-Kastler 2011	28/30	22/30	**28/30**	**25/30**	**2. Data did not match original trial.**
Domurath 2011	33/36	30/36	33/36	30/36	None.
Analysis 4.4					
Trials	Shorter length (number reporting preference/all subjects)	Standard length (number reporting preference/all subjects)	Shorter length (number reporting preference/all subjects)	Standard length (number reporting preference/all subjects)	
Chartier-Kastler 2013	67/112	45/112	67/112	45/112	None.
Costa 2013	7/81	74/81	7/81	74/81	None.
Domurath 2011	23/24	19/23	**22/36**	**14/36**	**2. Data did not match original trial.**
Biering-Sorensen 2007	22/36	14/36	**N/A**	**N/A**	**4. Data in form that could not be used in meta-analysis.**

Any information that differs between original trial data and 2014 Cochrane data is stated in **bolded blue font**.
There are five types of discrepancies in data extraction (right column, **bolded red font**):
1. Data mislabeled.
2. Data did not match original trial.
3. Data extracted in a method not consistent with convention.
4. Data in a form that could not be used in meta-analysis.
5. Data not originally extracted.
N/A, not applicable; SD, standard deviation; UTI, urinary tract infection.
