# Supplementary material for: Intermittent Catheterization: The Devil Is in the Details
Source: J Neurotrauma. 2018 Apr 1;35(7):985–9. doi: 10.1089/neu.2017.5413 (PMC5865623; doi:10.1089/neu.2017.5413)
Supplement: Supplemental data [file Supp_Fig1.pdf]

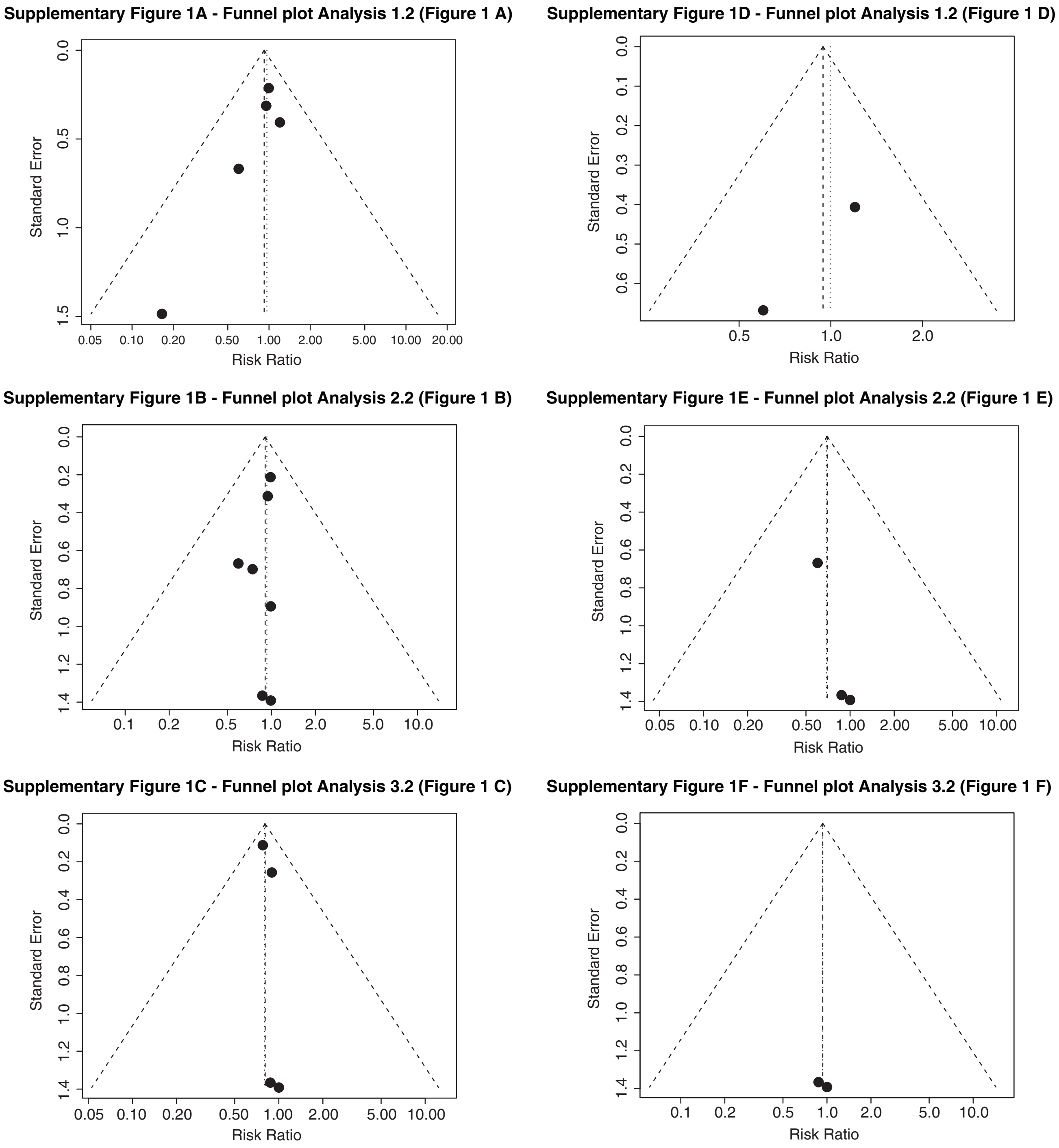


**SUPPLEMENTARY FIG. 1.**
